# Effects of LED Light Spectra on the Development, Phytochemical Profile, and Antioxidant Activity of *Curcuma longa* from Easter Island

**DOI:** 10.3390/plants11202701

**Published:** 2022-10-13

**Authors:** María José Marchant, Paula Molina, Miriam Montecinos, Leda Guzmán, Cristóbal Balada, Mónica Castro

**Affiliations:** 1 Laboratory of Biomedicine and Biocatalysis, Instituto de Química, Facultad de Ciencias, Pontificia Universidad Católica de Valparaíso, Avenida Universidad 330, Valparaíso 2340000, Chile; 2 Propagation Laboratory, Escuela de Agronomía, Facultad de Ciencias Agronómicas y de los Alimentos, Pontificia Universidad Católica de Valparaíso, La Palma S/N, Quillota 2260000, Chile

**Keywords:** turmeric, LED lighting, Rapa Nui, medicinal plants, secondary metabolites, radical scavenging activity

## Abstract

*Curcuma longa* (*C. longa*), an herbaceous plant used for medicinal purposes by the indigenous people of Easter Island, has been overexploited in its natural habitat, leading to its conservation status being designated as a vulnerable species. We have recently reported on the use of light-emitting diodes (LEDs) to improve the productivity of *C. longa* in vitro cultures under a temporary immersion system (TIS), but the effects of light quality on plant growth, phytochemical composition, and antioxidant capacity remained unexplored. Here, we set out to study these three aspects as observed at the end of TIS culture (day 0) and after 30 days of greenhouse acclimation (day 30). Thus, we evaluated plant morphological characteristics, phytochemical profile (polyphenols, tannins, flavonoids, reducing sugars, and curcumin), and radical scavenging activity by DPPH, ORAC, and FRAP assays. The results showed that, during in vitro cultivation under TIS, the red:blue (RB) LED light spectrum promoted *C. longa* shoot proliferation, with the resulting seedlings exhibiting greater fresh weight and no signs of etiolation. In the acclimation phase, the RB spectrum increased phytochemicals, such as polyphenols, flavonoids, and reducing sugars, and boosted curcumin synthesis. Nevertheless, the antioxidant activity of the plants under the RB light spectrum did not intensify. We surmise that this may be due to the premature intraplant allocation of metabolites to alternative pathways (e.g., curcumin synthesis) under RB light.

## 1. Introduction

*Curcuma longa* L. (*C. longa*) is an herbaceous plant widely used in traditional medicine, especially in China and India. In Chile, *C. longa* is mainly cultivated on Easter Island (also known as Rapa Nui), where it is used to make medicinal extracts to treat cancer, diabetes, muscle pain, etc. A characteristic of the ecotype of *C. longa* from Rapa Nui is its high concentration of polyphenols and curcumin. Therefore, it has been shown to have attractive biological activity properties, such as antioxidant, antimicrobial, anti-inflammatory, and anticancer activities [1,2]. However, *C. longa* (leaves and rhizomes) is overexploited, which has led to its conservation status being designated as a vulnerable species.

We recently reported a study on the in vitro micropropagation of *C. longa* using a temporary immersion system (TIS). In that study, we created experimental culture conditions with the goal of increasing in vitro production of *C. longa* under TIS. We demonstrated that factors such as the number of explants per flask, flask volume, and light quality all affected the in vitro growth of *C. longa*. The use of 30 explants per 2 L flask under LEDs with a red:blue ratio of 2:1 resulted in the highest number of seedlings (highest proliferation rate) [3]. However, the effect of light quality on the development, antioxidant capacity, and phytochemical profile of *C. longa* remained unclear.

Multiple plant growth factors, such as cytokinins, temperature, water, nutrients, and light quality, are responsible for the development, yield, and accumulation of phytochemical compounds in medicinal plants [4]. Environmental control by means of light-emitting diodes (LEDs) has been reported to have many advantages, including plant growth control, efficient energy conversion, long service life of lighting fixtures, low thermal energy output, and wavelength selectivity. Furthermore, recent studies have shown that the use of LEDs in seedling cultivation increases the concentration of bioactive compounds [5,6,7].

Light is an important environmental factor affecting phenological characteristics of plant development, such as growth rate, biomass production, and vigor. Several color wavelengths that make up visible light, in particular, red, blue, and green, have been found to have specific effects on plant growth rate, developmental characteristics, and production of bioactive compounds [8,9].

Illumination with LEDs offers great potential for in vitro seedling propagation and proliferation. The long service life, low thermal radiation, and energy efficiency of diodes make them ideal for in vitro cultivation [10]. The most important factor, however, is that they offer the possibility to fine-tune spectral compositions and tailor them to the specific needs of plants. The proper ratio of blue, red, and green wavelengths is the most important factor in plant growth, including plant morphogenesis and metabolism [11]. In addition, spectral tuning makes it possible to modify the synthesis pathways of primary and secondary metabolites in plants, and thus, to influence the accumulation of functional molecules [12]. Many studies have shown that total phenolic compounds and flavonoids increase when plants are exposed to the appropriate light spectrum during in vitro propagation [13,14]. However, the specific spectral composition required to enhance *C. longa* growth in vitro and boost metabolite production and accumulation has not yet been fully elucidated.

The purpose of this study was, therefore, to determine the effects of two different LED light qualities on the growth rate, phytochemical composition, and antioxidant activity of *C. longa*. The three parameters were first evaluated when the shoot growth of *C. longa* in vitro under TIS had terminated (day 0) and the seedlings were transferred to the greenhouse. The same parameters were then analyzed one month after the initiation of greenhouse acclimation (day 30) to determine whether the effects persisted over time.

## 2. Results

### 2.1. Effect of Light Quality on In Vitro Growth and Development of C. longa 

**Figure 1:**
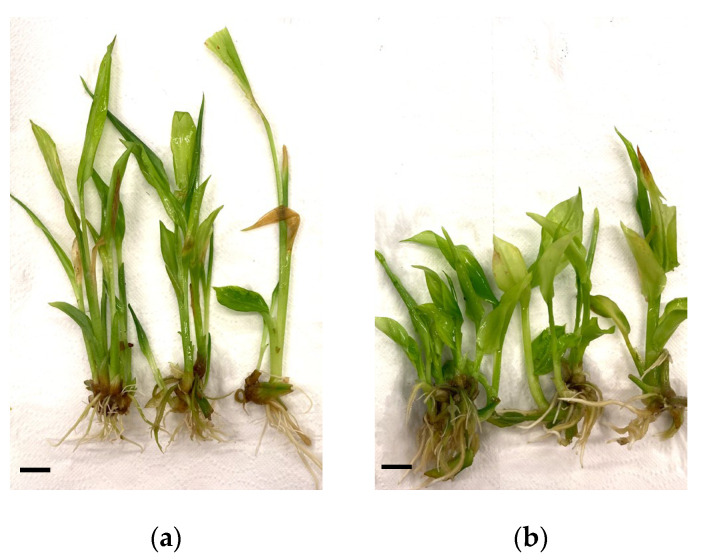
Effect of light quality on the development of *C. longa* after 45 days of in vitro culture. Plants irradiated with (**a**) white LED light and (**b**) RB LED light. The bar corresponds to 1 cm.

Plants produced in this study were propagated in vitro using TIS in mainland Chile, prior to shipment to Easter Island. Plants were fully acclimated in a greenhouse prior to shipment [3]. After 45 days of growth and rooting in vitro, *C. longa* plants were extracted, and their morphological characteristics were analyzed. A total of 284 *C. longa* plants were obtained from the white light treatment, and 311 from the RB light treatment. Plants from the white light treatment showed elongated leaves, fewer leaves per unit stem length, and larger pale and oxidized areas characteristic of etiolation. In addition, thin and short roots were observed (Figure 1a). In contrast, plants exposed to the RB light spectrum had uniform green pigmentation, less oxidation, and thicker and longer roots.

**Figure 2:**
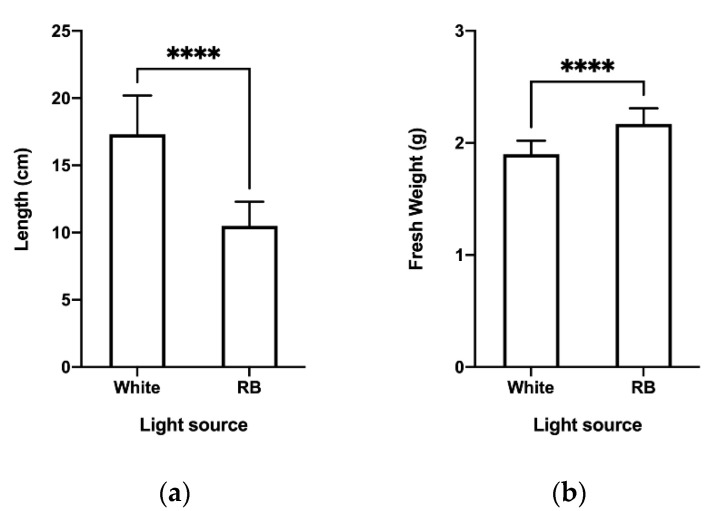
Effect of light quality on the length and fresh weight of *C. longa* plants at the end of TIS culture (day 0). (**a**) Plant length; (**b**) fresh weight per plant. cm: centimeter, g: gram. Mean ± SD were plotted for all replicates analyzed. Tukey’s multiple comparison tests were performed at a 95% confidence level in order to compare the differences. **** indicates a significant difference at *p* < 0.0001.

Furthermore, measurements of plant length and fresh weight at the end of TIS culture (Figure 2) revealed significant differences in both parameters: plants exposed to white light had greater height, but lower fresh weight compared to RB light-treated plants.

**Figure 3:**
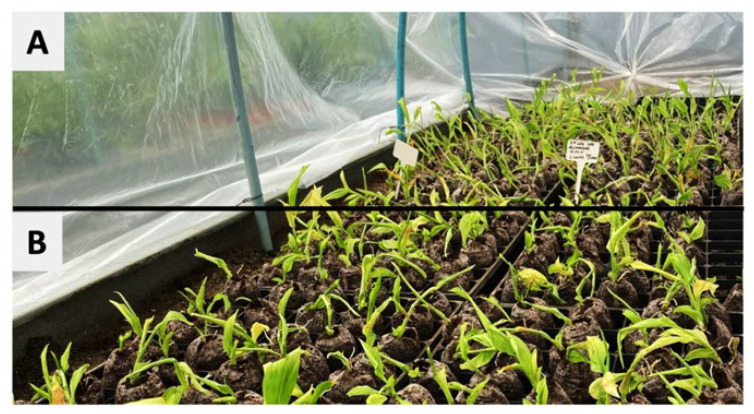
*C. longa* plants after 30 days of greenhouse acclimation. The upper part (**A**) shows plants exposed to RB LED light in in vitro propagation under TIS. The lower part (**B**) shows plants exposed to white LED light in in vitro propagation under TIS.

*C. longa* plants obtained by in vitro propagation using TIS under white and RB light wavelengths were subsequently acclimatized in a greenhouse under the same respective light spectra. Figure 3 shows the plants in the greenhouse after 30 days of acclimation. No morphological differences were observed in plants exposed to the two light qualities. Notably, plants exposed to RB light were shorter after TIS culture, but by the end of acclimation, were able to reach the same height as plants exposed to white light.

### 2.2. Effects of Light Quality on the Phytochemical Profile of C. longa

**Figure 4:**
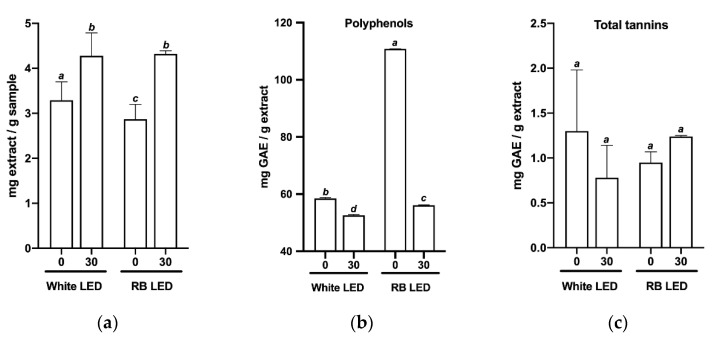

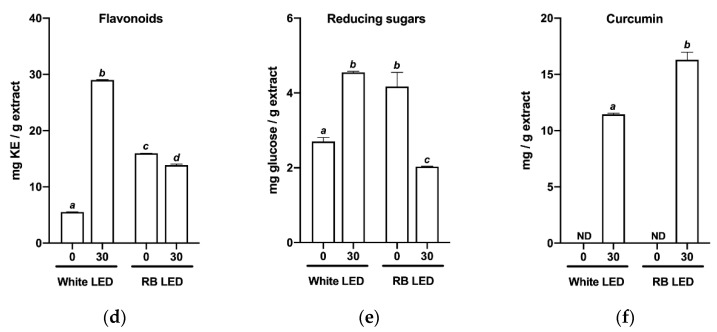
Phytochemical profiles of *C. longa* plants grown under different light spectra. Parameters were determined at the beginning of acclimation (0) and 30 days later (30). (**a**) Biomass (milligrams of extract per gram of plant matter); (**b**) polyphenols; (**c**) total tannins; (**d**) flavonoids; (**e**) reducing sugars; (**f**) curcumin. mg: milligrams, g: grams, GAE: gallic acid equivalents, KE: kaempferol equivalents, ND: not detected. Mean ± SD were plotted for all replicates analyzed. Tukey’s multiple comparison tests were performed at a 95% confidence level to compare the differences. Bars with different letters indicate significant differences at *p* < 0.05 for each parameter studied.

Concentrations of phytochemicals such as polyphenols, tannins, flavonoids, reducing sugars, and curcumin in *C. longa* plants were determined and compared for both treatments at the end of in vitro TIS culture (day 0) and at day 30 of subsequent greenhouse acclimation (Figure 4). The analysis showed that the phytochemical profiles of plants exposed to the two treatments were different, with plants under white light treatment initially having higher mg levels of extract per gram of plant matter compared to the RB light treatment. However, after 30 days of greenhouse acclimation under the same respective light spectra and in similar environmental conditions (Figure 4a), the difference disappeared.

At day 0, plants illuminated with white light had half the quantity of polyphenols, flavonoids, and reducing sugars as plants exposed to RB light. However, at day 30, plants in the white light treatment had significantly greater total tannins, reducing sugars, and curcumin, but fewer polyphenols.

Meanwhile, plants in the RB light treatment exhibited a significant decrease in polyphenols, flavonoids, and reducing sugars after 30 days in the greenhouse.

At the end of the TIS culture period, curcumin was not detected in plants grown under either treatment. However, after 30 days of greenhouse acclimation, both treatments produced curcumin, with RB light-treated plants having higher levels than plants grown under white light (Figure 4f). At the same time, no statistically significant differences were found with respect to total tannin content (Figure 4c).

### 2.3. Effect of Light Spectra on C. longa Radical Scavenging Activity 

The radical scavenging activity of *C. longa* plant extracts from the two treatments was determined by three separate methods: free radical scavenging activity of 2,2-diphenyl-1-picrylhydrazyl (DPPH), Oxygen Radical Absorbance Capacity (ORAC), and Ferric Reducing Ability of Plasma (FRAP). These are conventional methods for measuring the radical scavenging activity of various substances. Vitamin C (VIT C), Trolox, and gallic acid (GA) were used as positive controls in the DPPH, ORAC, and FRAP assays, respectively.

**Figure 5:**
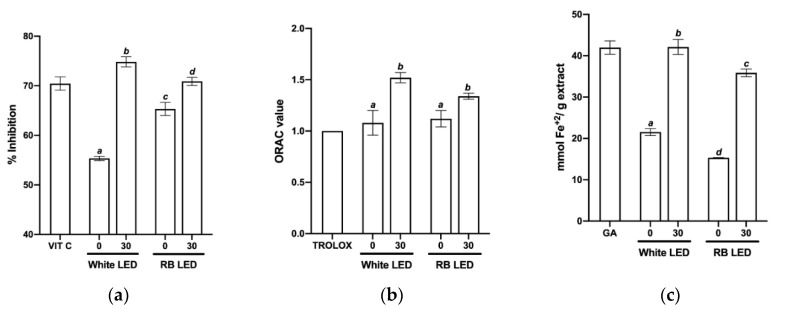
Radical scavenging activity of *C. longa* plants grown under different light spectra, at the beginning of acclimation (0) and after 30 days (30) in the greenhouse. (**a**) DPPH assay; (**b**) ORAC assay; (**c**) FRAP assay. Mean ± SD were plotted for all replicates analyzed. Vitamin C (VIT C), Trolox, and gallic acid (GA) were used as positive controls in the DPPH, ORAC, and FRAP assays, respectively. Tukey’s multiple comparison tests were performed at a 95% confidence level to compare the differences between treatments, for each parameter analyzed. Bars with different letters indicate significant differences at *p* < 0.05.

Figure 5 shows the results of the DPPH, ORAC, and FRAP assays, as well as measurements of the radical scavenging capacity of the extracts evaluated. At the beginning of greenhouse acclimation (day 0), plants exposed to RB light showed higher DPPH scavenging activity compared to plants exposed to white light. After 30 days, the trend was reversed (Figure 5a).

According to the ORAC assay, plants in both treatments initially showed similar radical scavenging activity, but after 30 days in the greenhouse, the activity of white light-treated plants was slightly higher (Figure 5b).

The largest difference was observed in the FRAP assay, however, where white light-treated plants showed greater ferric reducing capacity both at the start of acclimation and after 30 days in the greenhouse (Figure 5c).

## 3. Discussion

We have previously reported experimental conditions for in vitro propagation in TIS culture that would increase production of the Easter Island *C. longa* ecotype [3]. However, improving the quality of the resulting seedlings would also be very important. Light plays a key role in the processes of photosynthesis and energy conversion in plants. Analysis of the spectral composition of LED luminaires used in this study revealed that the RB and white light spectra differed in their proportions of red, green, blue, and far-red photons (Table 1). The most significant differences were observed in the percentages of green and red photons: 40.7% and 26.2% in white light compared to 0.5% and 68.4% in RB light, respectively. Furthermore, RB light provided higher daily photosynthetically active irradiance (daily light integral, DLI). These differences affected the growth rate, phytochemical composition, and antioxidant activity of *C. longa* propagated in vitro.

We first evaluated in vitro growth of *C. longa* shoots under two different light spectra. Red and blue wavelengths provide energy to activate photoreceptors and trigger a series of metabolic processes during plant growth, including stem elongation, leaf expansion, stomatal opening, circadian clock, and flowering [15,16]. The RB LED light increased the production of phytochemicals that are used during the growth of *C. longa*. However, we found that RB LED light promoted the development of more compact seedlings with greater fresh weight, and with leaves without signs of etiolation or chlorosis.

In contrast, seedlings exposed to white light exhibited etiolation, a phenomenon in which morphological changes (elongation of leaves and stems) are activated by the inadequate spectral composition of light [17]. The white light spectrum used in this study had a high proportion of green and far-red photons. This combination has been reported to trigger more shade-induced traits than green or far-red wavelengths alone [18]. In addition, signs of chlorosis (low chlorophyll production) were observed in the leaves, leading to lower synthesis of reducing sugars and other carbohydrates in white light-treated plants [19].

Specific light spectra can alter phytochemical profiles and metabolite accumulation rates in different plant parts (e.g., leaves, rhizomes, and stems). Several authors have shown that certain proportions of red, blue, and green wavelengths in LED luminaires improve growth and development, nutrient levels, and the production and accumulation of bioactive compounds [8,9,20]. Appropriately selected spectral compositions have indirect regulatory effects on gene expression related to metabolite synthesis that help plants adapt to changes in lighting [15].

During in vitro propagation, shoot concentrations of polyphenols, flavonoids, and reducing sugars were found to be higher under RB illumination compared to white light. However, after 30 days in the greenhouse, these analytes decreased in RB light-treated plants, while curcumin levels increased compared to the white light-treated plants. Thus, the proportion of red photons in RB light seems to be the key to gene expression associated with the synthesis of flavonoids and other antioxidants [21,22].

Indeed, similar results were obtained in other species, where exposure to LED lighting with different red:blue ratios increased both antioxidant capacity and polyphenols [23]. Klimek-Szczykutowicz [24] obtained results consistent with the results of the present study when biomass, reducing sugars, and polyphenols, as well as DPPH and FRAP radical scavenging activity, were determined in *Nasturtium officinale* samples exposed in vitro to LED lighting with different spectral compositions. The concentration of polyphenols and reducing sugars was observed to increase under illumination dominated by blue and red photons [24]. Thus, further studies on the expression of genes related to metabolite production under different light stimuli, as well as its dynamics over time, would be very informative.

Finally, extracts of *C. longa* from the two treatments were evaluated for their antioxidant activity. Different light spectra can affect the production of reactive oxygen species (ROS), induce oxidative damage, and inhibit the synthesis of secondary metabolites [25]. Studies show that exposure to blue light increases the radical scavenging activity of plant extracts through enhanced synthesis of secondary metabolites, such as anthocyanins, flavonoids, and polyphenols [8,26,27,28].

Another important observation was the contrasts between the three antioxidant activity assays. White light-treated plants showed higher radical scavenging activity than plants treated with RB light, even though the latter had a higher concentration of polyphenols and accumulated more curcumin. Curcumin is a potent radical scavenger [29], but it accumulated within the rhizomes during the greenhouse acclimation phase and was diluted with components from other parts of the plant during extraction.

In addition, the efficiency of phenolics extraction depends on the method used, as the composition and antioxidant capacity of the obtained components may vary [30]. At the end of TIS culture, RB light-treated plants showed higher antioxidant capacity in DPPH and ORAC assays compared to white light-treated plants. However, after 30 days in the greenhouse, white light-treated seedlings exhibited greater antioxidant capacity in DPPH and FRAP assays, even exceeding that of various positive controls.

The red:blue light treatment in TIS culture increased the synthesis of antioxidant compounds, such as polyphenols and flavonoids, as well as reducing sugars, which became readily available to plants for use in curcumin synthesis during the acclimation phase [31,32]. Premature intraplant allocation of these compounds to alternative metabolic pathways during greenhouse acclimation may be responsible for the reduced antioxidant activity and exaggerated curcumin accumulation in RB light-treated plants.

## 4. Materials and Methods

### 4.1. Explant Collection, Disinfection, and Establishment of Rhizome Cultures 

*C. longa* rhizomes were harvested from the Mataveri Otai nursery on Easter Island (geographical coordinates: 27°09′50.17″ S–109°26′24.63″ W). The excised long creeping rhizomes were thoroughly washed with water and Tween-20 for 10 min. The rhizomes were then cut into 2–3 cm pieces and placed under running water for 30 min. Subsequently, the rhizomes were immersed in the Phyton solution (3 mL/L) under gentle agitation for 15 min. Next, the rhizomes were cut into 1 cm pieces, and active buds were separated and washed with 1.5% sodium hypochlorite solution, 0.5 g/L citric acid, and 0.5 g/L ascorbic acid for 15 min under constant agitation. The pieces were transferred to a laminar flow hood and washed four times with a sterile solution of 400 mg/L citric acid and 400 mg/L ascorbic acid. Finally, the pieces were placed in a Murashige and Skoog (MS) culture medium supplemented with 0.1 mg/L thiamine, 200 mg/L glycine, 170 mg/L NaH_2_PO_4_, 2.5 mL/L plant preservative mixture (PPM), 6.5 g/L agar, and 30 g/L sucrose at pH 5.8. The flasks were incubated in vitro in the dark at 25 °C for 10 days, and then placed under 1R/1B LED light (50% red (peak at 660 nm) + 50% blue (420–460 nm), PARALED system, Ciencia Pura SpA, Santiago, Chile) for one month.

### 4.2. In Vitro Growth of Explants

The shoot rhizomes were transferred to the MS growth medium, supplemented with 0.1 mg/L thiamine, 200 mg/L glycine, 170 mg/L NaH_2_PO_4_, 1 mg/L 1-Naphthaleneacetic acid (NAA), 2 mg/L N_6_-Benzylaminopurine (BAP), 6.5 g/L agar, and 30 g/L sucrose at pH 5.8. The plant material was subcultured three times (30 days each time) to obtain more shoots for TIS. The initial explants used for TIS were 1–2 cm long, rootless, and showed no symptoms of vitrification (hyperhydricity) or oxidation.

### 4.3. In Vitro Proliferation of Curcuma longa by TIS

A temporary immersion system was used for the proliferation and rooting of *C. longa* shoots. Proliferation was performed in an MS culture medium, supplemented with 200 mg/L glycine, 0.1 mg/L thiamine, 0.5 mg/L nicotinic acid, 0.5 mg/L pyridoxine, 100 mg/L myoinositol, 170 mg/L NaH_2_PO_4_, 3 mg/L BAP, and 30 g/L sucrose at pH 5.8. For the rooting stage, the same medium was used, and BAP was replaced with 1 mg/L NAA. Twenty-one flasks for each set of conditions were used. Flasks were maintained under the following conditions during proliferation and rooting: 30 explants and 450 mL of culture medium (proliferation or rooting) per flask, immersion of 4 min each time in a 4 h cycle, and a temperature of 23 ± 1 °C for 16 h/day. After the proliferation stage, the flask of the proliferation medium was replaced with the flask of the rooting medium under a laminar flow hood, while the explants were not disturbed by handling. After both stages, all shoots were placed in a greenhouse to acclimate. The light and environmental conditions were the same for all the conditions studied.

### 4.4. Spectral Light Conditions

To explore the effects of LED light on the growth and phytochemical composition of *C. longa*, two treatments were set up as follows: (i) white LED light as control; and (ii) RB LED light (PARALED system, Ciencia Pura SpA, Santiago, Chile). Photon fluxes were measured using a compact spectrophotometer (Lighting Passport Standard Pro model, Allied Scientific ProTM, Gatineau, QC, Canada). To avoid light contamination, all assays were separated from each other by white screens made by a local manufacturer. The characteristics of experimental illumination are shown in Table 1.

### 4.5. Phytochemical Composition

Samples of *C. longa* plants were collected at the end of TIS culture and on day 30 after the initiation of acclimation. Samples were processed and analyzed to determine the amounts of the following metabolites: polyphenols, tannins, flavonoids, reducing sugars, and curcumin. Samples were dried at 40 °C for two weeks, and lipids were extracted using hexane solvent with a Soxhlet system.

#### 4.5.1. Polyphenols

To obtain polyphenol-rich extracts, dry samples were mixed with 95% ethanol under agitation for 48 h. The ethanol was removed with a rotary evaporator and the extracts were resuspended in DMSO. A modified Folin–Ciocalteu method was used to quantify the total content of polyphenols [33]. The results were expressed in mg gallic acid equivalent (GAE)/g extract.

#### 4.5.2. Total Tannins

A modified Folin–Ciocalteu method was used for the quantification of tannins [33]. Briefly, 100 µL extract and 500 µL water were mixed with 250 μL Folin–Ciocalteu 1N reagent under vigorous shaking. Then, 35% Na_2_CO_3_ was added, and the sample was shaken again for 30 min. The absorbance was measured at 725 nm and the data were interpolated on the gallic acid calibration curve. The results were expressed in mg gallic acid equivalent (GAE)/g extract.

#### 4.5.3. Flavonoids

Flavonoids were determined by the method of Liu [34], with modifications. The reaction mixture contained 30 µL sodium nitrite (10% *w*/*v*), 60 µL aluminum chloride hexahydrate (20% *w*/*v*), 200 µL NaOH (1M), 400 µL distilled water, and a 100 µL sample for analysis. The absorbance was measured after 5 min of reaction at 415 nm, and the values were interpolated on a kaempferol calibration curve. The results were expressed as mg kaempferol (KE) per gram of dry extract. The experiments were performed in triplicate.

#### 4.5.4. Reducing Sugars

Reducing sugars were quantified by the modified DNS method [35], whereby 100 µL extract and 1000 µL DNS reagent were mixed with vortex; the mixture was heated in boiling water for 5 min; then, 8 mL water was added, and the absorbance was measured at 540 nm. The results were expressed in mg glucose/L.

#### 4.5.5. Determination of Curcumin

A calibration curve of 1 to 25 μg/mL curcumin was made, and the absorbance was measured at 421 nm. Dilutions of the original extract were made and measured at 421 nm, according to the modified protocol of Hazra et al. [36].

### 4.6. Radical Scavenging Capacity

#### 4.6.1. DPPH Assay

The radical scavenging activity of the extracts was evaluated by the DPPH (1,1-diphenyl-2-picrylhydrazyl) assay [37]. Briefly, 1 mL of 0.1 mM DPPH radical solution in ethanol was mixed with 50 μL leaf, rhizome extracts, gallic acid, or vitamin C at a concentration of 20 μg/mL. The antioxidant activity of the extracts was evaluated by the DPPH assay. DPPH was reduced and the color change was measured at 518 nm after 20 min of reaction time using an Epoch ELISA reader (ELx800, BioTek^®^, Winooski, VT, USA). The percentage of DPPH inhibition was calculated using the following equation:

(1) $$\%\;\mathrm{Inhibition}=\left(\frac{\mathrm{Abs}\;\mathrm{control}-\mathrm{Abs}\;\mathrm{sample}}{\mathrm{Abs}\;\mathrm{control}}\right)\times 100$$

where “Abs control” is the absorbance of DPPH in the absence of a sample, and “Abs sample” is the absorbance of DPPH in the presence of either a sample or the standard (gallic acid). The experiments were performed in triplicate.

#### 4.6.2. ORAC-FL Assay

The ORAC value was measured according to the method described by Ou [38], with modifications [39]. The reaction was conducted in sodium phosphate buffer (75 mM, pH 7.4) using black-walled 96-well plates with a final volume of 200 µL. Twenty µL each of extract or Trolox (20 μg/mL) and fluorescein solutions (120 µL; 70 nM, final concentration) were placed in each microplate well. The mixture was pre-incubated for 15 min at 37 °C. The AAPH solution (60 μL; 12 mM final concentration) was added rapidly, and the microplate was read immediately with a fluorescence reader (Synergy HT multi-detection microplate reader; BioTek^®^ Instruments, Inc., Winooski, VT, USA). Fluorescence was recorded every minute for 80 min from normalized curves, and the area under the fluorescence decay curve (AUC) was calculated as follows:

(2) $$AUC=1+\overset{i=80}{\underset{i=1}{\sum }}\frac{f_{i}}{f_{0}}$$

where *f*_0_ is the initial fluorescence reading at 0 min and *f_i_* is the fluorescence reading at time *i*. The AUC of a sample was calculated by subtracting the AUC of the blank space. Regression equations between net AUC and antioxidant concentration were calculated for all samples. ORAC-FL values were expressed as Trolox equivalents using the standard curve calculated for each assay. Results were expressed in µmol of Trolox equivalent/µmol of extract. The experiments were performed in triplicate.

#### 4.6.3. FRAP Assay

The iron reducing power of each fraction was determined as described by Dudonné [40], with modifications. A functional FRAP reagent was prepared freshly by mixing 10 volumes of 300 mM acetate buffer, pH 3.6, 1.0 volume of 10 mM TPTZ (2,4,6-tri(2-pyridyl)-s-triazine) in 40 mM hydrochloric acid, and 1.0 volume of 20 mM ferric chloride; 100 µL sample solution (1 mg/mL) and 300 µL deionized water were added to 3 mL of the freshly prepared FRAP reagent. The reaction mixture was incubated for 30 min at 37 °C in a water bath. The absorbance of the samples was then measured at 593 nm, and FRAP values were expressed as mmol Fe^2+^/g sample. All measurements were performed in triplicate.

### 4.7. Statistical Analysis

A two-way ANOVA, along with Tukey’s multiple comparison tests, was used for comparisons; *p* < 0.05 was considered a statistically significant difference. All statistical analyses were calculated using the GraphPad Prism 9 computer software (GraphPad Software, San Diego, CA, USA).

## 5. Conclusions

Red:blue LED light enhanced the proliferation of *C. longa* shoots in vitro by TIS. In addition, the resulting plantlets had greater fresh weight and no signs of etiolation. Plants under RB light also exhibited higher levels of phytochemicals such as polyphenols, flavonoids, and reducing sugars, which stimulated curcumin synthesis in the acclimation phase. Nevertheless, RB light-treated plants did not exhibit better antioxidant activity, possibly due to the premature intraplant allocation of metabolites to alternative pathways, such as curcumin synthesis. The molecular mechanism underlying these regulatory processes is unclear and warrants further investigation.

## Figures and Tables

**Table 1:** Spectral distribution of light used in the experiment. RB: red:blue, m: meter, DLI: daily light integral.

Parameter	Spectral Fraction of Light Source (%)	
	White	RB
Photon flux (380–780 nm)	100 ^a^	100 ^a^
Ultraviolet light (380–399 nm)	0.1	0.1
Blue light (400–499 nm)	32.3	30.9
Green light (500–599 nm)	40.7	0.5
Red light (600–700 nm)	26.2	68.4
Far-red light (701–780 nm)	0.7	0.1
DLI (mol m^−2^)	0.86	3.80
R:B ratio ^b^	0.63	1.74
R:FR ratio ^c^	8.41	113.20
Light source	White LED light	LED with red and blue chips

^a^ Data are fractions of YPDF ranging from 380 to 780 nm in ultraviolet, blue, green, red, and far-red light. ^b^ R:B ratio is an abbreviation for the ratio of red light to blue light. ^c^ R:FR ratio is an abbreviation for the ratio of red light to far-red light.

## References

[1] Sharifi-Rad J., El Rayess Y., Rizk A.A., Sadaka C., Zgheib R., Zam W., Sestito S., Rapposelli S., Neffe-Skocińska K., Zielińska D. (2020). Turmeric and Its Major Compound Curcumin on Health: Bioactive Effects and Safety Profiles for Food, Pharmaceutical, Biotechnological and Medicinal Applications. Front. Pharmacol..

[2] Balada C., Castro M., Fassio C., Zamora A., Marchant M.J., Acevedo W., Guzmán L. (2021). Genetic diversity and biological activity of Curcuma longa ecotypes from Rapa Nui using molecular markers. Saudi J. Biol. Sci..

[3] Marchant M.J., Molina P., Montecinos M., Guzmán L., Balada C., Fassio C., Castro M. (2021). In Vitro Propagation of Easter Island *Curcuma longa* from Rhizome Explants Using Temporary Immersion System. Agronomy.

[4] Al Murad M., Razi K., Jeong B., Samy P., Muneer S. (2021). Light Emitting Diodes (LEDs) as Agricultural Lighting: Impact and Its Potential on Improving Physiology, Flowering, and Secondary Metabolites of Crops. Sustainability.

[5] Le A.T., Yu J.-K., Han G.-D., Do T.K., Chung Y.-S. (2022). Potential Use of Colored LED Lights to Increase the Production of Bioactive Metabolites *Hedyotis corymbosa* (L.) Lam. Plants.

[6] Cioć M., Szewczyk A., Żupnik M., Kalisz A., Pawłowska B. (2018). LED lighting affects plant growth, morphogenesis and phytochemical contents of Myrtus communis L. in vitro. Plant Cell Tissue Organ Cult. PCTOC.

[7] Azad M.O.K., Kjaer K.H., Adnan M., Naznin M.T., Lim J.D., Sung I.J., Park C.H., Lim Y.S. (2020). The Evaluation of Growth Performance, Photosynthetic Capacity, and Primary and Secondary Metabolite Content of Leaf Lettuce Grown under Limited Irradiation of Blue and Red LED Light in an Urban Plant Factory. Agriculture.

[8] Samuolienė G., Brazaitytė A., Sirtautas R., Novičkovas A., Duchovskis P. (2012). The Effect of Supplementary LED Lighting on the Antioxidant and Nutritional Properties of Lettuce. Acta Hortic..

[9] Dong C., Fu Y., Liu G., Liu H. (2014). Growth, Photosynthetic Characteristics, Antioxidant Capacity and Biomass Yield and Quality of Wheat (*Triticum aestivum* L.) Exposed to LED Light Sources with Different Spectra Combinations. J. Agron. Crop Sci..

[10] Alvarenga I.C.A., Pacheco F.V., Silva S.T., Bertolucci S.K.V., Pinto J.E.B.P. (2015). In vitro culture of Achillea millefolium L.: Quality and intensity of light on growth and production of volatiles. Plant Cell Tissue Organ Cult. PCTOC.

[11] Li H., Tang C., Xu Z. (2013). The effects of different light qualities on rapeseed (*Brassica napus* L.) plantlet growth and morphogenesis in vitro. Sci. Hortic..

[12] Darko É., Heydarizadeh P., Schoefs B., Sabzalian M.R. (2014). Photosynthesis under artificial light: The shift in primary and secondary metabolism. Philos. Trans. R. Soc. B Biol. Sci..

[13] Jung W.-S., Chung I.-M., Hwang M., Kim S.-H., Yu C., Ghimire B. (2021). Application of Light-Emitting Diodes for Improving the Nutritional Quality and Bioactive Compound Levels of Some Crops and Medicinal Plants. Molecules.

[14] Hasan M., Bashir T., Ghosh R., Lee S.K., Bae H. (2017). An Overview of LEDs’ Effects on the Production of Bioactive Compounds and Crop Quality. Molecules.

[15] Chen M., Chory J., Fankhauser C. (2004). Light Signal Transduction in Higher Plants. Annu. Rev. Genet..

[16] Su J., Liu B., Liao J., Yang Z., Lin C., Oka Y. (2017). Coordination of Cryptochrome and Phytochrome Signals in the Regulation of Plant Light Responses. Agronomy.

[17] Agrios G., Breibelbis D., Sonnack K.D., Beattie L., DeCicco E. (2005). Enviromental Factors That Cause Plant Diseases. Plant Pathology.

[18] Wang Y., Folta K.M. (2013). Contributions of green light to plant growth and development. Am. J. Bot..

[19] Pham M.D., Hwang H., Park S.W., Cui M., Lee H., Chun C. (2019). Leaf chlorosis, epinasty, carbohydrate contents and growth of tomato show different responses to the red/blue wavelength ratio under continuous light. Plant Physiol. Biochem..

[20] Bian Z.H., Yang Q.C., Liu W.K. (2015). Effects of light quality on the accumulation of phytochemicals in vegetables produced in controlled environments: A review. J. Sci. Food Agric..

[21] Dutta Gupta S., Pradhan S., Dutta Gupta S. (2017). Regulation of Gene Expression by LED Lighting. Light Emitting Diodes for Agriculture: Smart Lighting.

[22] Meng X., Xing T., Wang X. (2004). The role of light in the regulation of anthocyanin accumulation in Gerbera hybrida. Plant Growth Regul..

[23] Ahmadi T., Shabani L., Sabzalian M.R. (2020). LED light mediates phenolic accumulation and enhances antioxidant activity in Melissa officinalis L. under drought stress condition. Protoplasma.

[24] Klimek-Szczykutowicz M., Prokopiuk B., Dziurka K., Pawłowska B., Ekiert H., Szopa A. (2022). The influence of different wavelengths of LED light on the production of glucosinolates and phenolic compounds and the antioxidant potential in in vitro cultures of Nasturtium officinale (watercress). Plant Cell Tissue Organ Cult..

[25] Lü J.-M., Lin P.H., Yao Q., Chen C. (2010). Chemical and molecular mechanisms of antioxidants: Experimental approaches and model systems. J. Cell. Mol. Med..

[26] Kim K., Kook H.-S., Jang Y.-J., Lee W.-H., Kamala-Kannan S., Chae J.-C., Lee K.-J. (2013). The Effect of Blue-light-emitting Diodes on Antioxidant Properties and Resistance to Botrytis cinerea in Tomato. J. Plant Pathol. Microbiol..

[27] Lee S.-C., Kim J.-H., Jeong S.-M., Kim D.-R., Ha J.-U., Nam K.C., Ahn D.U. (2003). Effect of Far-Infrared Radiation on the Antioxidant Activity of Rice Hulls. J. Agric. Food Chem..

[28] Alrifai O., Hao X., Marcone M.F., Tsao R. (2019). Current Review of the Modulatory Effects of LED Lights on Photosynthesis of Secondary Metabolites and Future Perspectives of Microgreen Vegetables. J. Agric. Food Chem..

[29] Borra S.K., Gurumurthy P., Mahendra J., Jayamathi K.M., Cherian C.N., Chand R. (2013). Antioxidant and free radical scavenging activity of curcumin determined by using different in vitro and ex vivo models. J. Med. Plant Res..

[30] Kim G.-H., Duan Y., Kim H.-S. (2016). Effects of Various Extracts from Turmeric (*Curcuma longa* L.) on Antioxidant Activity. J. Korean Oil Chem. Soc..

[31] Chouhan S., Sharma K., Zha J., Guleria S., Koffas M.A.G. (2017). Recent Advances in the Recombinant Biosynthesis of Polyphenols. Front. Microbiol..

[32] Sharangi A., Gowda M.P., Das S. (2022). Responses of turmeric to light intensities and nutrients in a forest ecosystem: Retrospective insight. Trees For. People.

[33] Ricco R.A., Agudelo I.J., Wagner M.L. (2015). Métodos Empleados En El Análisis de Los Polifenoles En Un Laboratorio de Baja Complejidad. Lilloa.

[34] Liu H., Song Y., Zhang X. (2017). Determination of total flavonoids in leek by alcl3 colorimetric assay. Chem. Eng. Trans..

[35] Gusakov A.V., Kondratyeva E.G., Sinitsyn A.P. (2011). Comparison of Two Methods for Assaying Reducing Sugars in the Determination of Carbohydrase Activities. Int. J. Anal. Chem..

[36] Hazra K., Kumar R., Kumar Sarkar B., Chowdary Y.A., Devgan M., Ramaiah M., Educational R.P. (2015). Uv-Visible Spectrophotometric Estimation of Curcumin in Nano-Formulation. Int. J. Pharma. Cognosy.

[37] Brand-Williams W., Cuvelier M.E., Berset C. (1995). Use of a free radical method to evaluate antioxidant activity. LWT Food Sci. Technol..

[38] Ou B., Hampsch-Woodill M., Prior R.L. (2001). Development and Validation of an Improved Oxygen Radical Absorbance Capacity Assay Using Fluorescein as the Fluorescent Probe. J. Agric. Food Chem..

[39] Dávalos A., Gómez-Cordovés A.C., Bartolomé B. (2004). Extending Applicability of the Oxygen Radical Absorbance Capacity (ORAC−Fluorescein) Assay. J. Agric. Food Chem..

[40] Dudonné S., Vitrac X., Coutière P., Woillez M., Mérillon J.-M. (2009). Comparative study of antioxidant properties and total phenolic content of 30 plant extracts of industrial interest using DPPH, ABTS, FRAP, SOD, and ORAC assays. J. Agric. Food Chem..

